# How the Establishment of the National Civilized City Promotes Urban Green Development: From the Perspective of Administrative Competing Theory—A Quasi Experiment Study in China

**DOI:** 10.3390/ijerph191711103

**Published:** 2022-09-05

**Authors:** Rongrong Shi, Dian Song, Guoqiang Rui, Hainan Wu

**Affiliations:** 1 Business School, Soochow University, Suzhou 215021, China; 2 School of Political and Public Administration, Soochow University, Suzhou 215021, China; 3 School of Finance and Public Administration, Anhui University of Finance & Economics, Bengbu 233030, China

**Keywords:** establishing national civilized city, green total factor productivity, administrative competing system, PSM–DID

## Abstract

Green development is the core goal for contemporary urban areas. It has become essential to explore new types of urban green development, and the concept of the “national civilized city” which is the most influential city brand in China, has emerged. Drawing upon the administrative competing theory, based on the panel data of 281 cities in China from 2000 to 2018, this paper employs a propensity scores matching (PSM) design and a difference-in-difference (DID) approach to examine the influence of the establishment of the national civilized city policy on the green development of prefecture-level cities in China. First, the result shows that the establishment of the national civilized city policy can significantly improve the urban green total factor productivity (GTFP). Second, the mediation mechanism analyses show that the establishment of national civilized city policy can promote cities to increase their investment in R&D, increase the financial investment in environmental governance, and optimize the industrial structure, which further lays a solid foundation for urban green development. Third, the heterogeneity analysis shows that the impact of civilized city construction on urban GTFP is different in terms of population and economic scales. The results show that the weight of environmental management and R&D investment should be increased in the evaluation index of the national civilized city, and the promotion of urban green development should be maximized.

## 1. Introduction

Since the reform and opening-up, China’s urbanization level has rapidly improved, and the urbanization rate exceeded 60% in 2019 [1,2]. An increasing number of Chinese cities have become global cities, and cities have become major engines of national development [3]. However, cities are an important carrier of national modernization and the places where urban diseases often occur [4]. Rapid urbanization leads to drawbacks such as air pollution [5,6], carbon emissions [7,8], traffic congestion [2,9], and huge resource consumption [9,10]. Facing these situations, China has designed a new urbanization strategy that underlines people-oriented environmental protection [11]. In 2017, the Chinese government stressed that a strict ecological and environmental protection system would be implemented and green development would be prioritized at an unprecedented level [12,13]. To maintain the balance between economic development and environmental protection, cities need to not only take measures to promote economic growth but also address environmental problems [14]. Therefore, many Chinese cities have adopted the green development model to facilitate green transformation [15].

Green total factor productivity (GTFP) is the core of urban green development [12,16], which is used to represent the development level of China’s green economy in the literature [12]. GTFP adds resource input and environmental impact indicators within the traditional total factor productivity (TFP) evaluation framework and can measure economic benefits at the cost of energy consumption and environmental pollution [17,18]. Therefore, GTFP considers the impact of economic activity on both resources and the environment [16]. Improving cities’ GTFP is directly related to the success of China’s transformation into a low-carbon, efficient and energy-saving economy [19,20].

Researchers have shown that human capital [21], environment regulation [22], agglomeration economic [23], foreign trade [24], and digital economy development [12] significantly impact a city’s GTFP. However, these factors are static indicators that cannot fully describe the government’s endeavor to enhance GTFP. Therefore, some scholars have explored what kinds of practices can enhance a city’s GTFP. In China, a large amount of attention has been paid to various types of city governance practices, such as establishing an innovative pilot city [25,26,27], constructing a low carbon pilot city [28,29], and building a smart city [2,30], whose facilitating effects on GTFP are validated.

Recently, theorists have begun to pay attention to the impact of the establishment of the national civilized city, which is most influential city brand practice in China, in terms of urban green development [31,32]. The aim of establishing a national civilized city in China is to construct a new model of sustainable urbanization with Chinese characteristics, which is conducive to building a more civilized and harmonious society, and effectively changing the mode of urban development [33]. Studies have shown that the establishment of national civilized cities can significantly reduce the environmental performance of firms [33], and reduce urban emissions by providing political incentives to promote officials to strengthen the management of pollution sources and introduce green enterprises [32,34]. In contrast, some studies have shown that the establishment of civilized cities significantly inhibits the human capital accumulation of firms and have a negative effect on economic growth in low-ranking cities [35], and decreases the growth rate of tourism resources and increases the financial burden [36].

In sum, the conclusions on the impact of establishing a national civilized city on urban green development are mixed, and more evidence is needed. Although previous studies have analyzed the direct impact of building a national civilized city on urban green development, few studies have elaborated on the impact mechanism of national civilized city construction on urban green development. Therefore, applying an administrative competing system as a research framework, this paper highlights that the reputation incentives, political incentives, and competitive incentives brought about by the establishment of national civilized cities are the driving mechanisms to encourage local governments to maintain a balance between economic growth and environmental governance compatibility, increasing R&D investment, strengthening environmental management infrastructure and promoting industrial upgrading are the key measures taken by cities in the process of the establishment of national civilized cities. Then, the research employs propensity score matching and difference-in-difference methods to test the above view and describes the functional principle and distinctive law of the establishment of national civilized cities as an administrative competing system arrangement, which enriches the theoretical research of national governance with Chinese characteristics [37].

## 2. Theoretical Background and Hypothesis Development

### 2.1. Administrative Competing System (ACS)

In the previous literature on government governance models, many theories, such as fiscal federalism, project system, promotion tournament theory, administrative competing system, and pressure system, assume that the central government adopts the same incentive mechanism and assessment standards as local governments, without taking into account the differences in conditions among local governments. In contrast to these theories, which mainly punish those that progress at a slower rate, the administrative competing system emphasizes positive incentives [37,38,39]. The administrative competing system is a city governance model with Chinese characteristics. It refers to the central government setting up various honorary city titles; designing clear, dynamic, high-standard indicators, and evaluation thresholds; and encouraging local governments to voluntarily and actively respond to the initiatives of central governments to participate in the honorary title declaration based on the city’s political, economic, and cultural conditions. Cities that pass the appraisal will be rewarded corresponding honorary titles, but the honorary titles will be retracted if the city fails to pass its reexamination; that is, the honorary title is not a lifelong title. The administrative competing system is beneficial for the central government to achieve its governance goals within the scope of its responsibility [37]. The policy targets of the administrative competing system are often the weak points or pain points in national governance. The goal of this is to achieve a partial breakthrough in governance performance in a certain field in a short period of time, and to commend cities that meet the standards, so as to form a demonstration effect [37].

First, ACS is conducive to local autonomy and the selection of an appropriate governance mode. ACS can be applied within a wide range of fields, such as urban health, landscaping, safety production, civilization, and smart management. The administrative competing system allows local governments to expand their investment in a certain field according to their own conditions, forming city brands with different positioning, such as a sanitary city, forest city, ecological garden city, civilized city and livable city, and providing local governments complete autonomy in choosing their governance mode. Cities are often more willing to declare ACS projects designed by departments with a high political status and strong political authority.

Second, ACS emphasizes positive incentives. If a city passes the examination, the central government only awards it the appropriate honorary title, which will not have obvious economic benefits or significantly improve the promotion opportunities of the city’s chief administrative officials. Meanwhile, if the city fails to pass the examination, the city will not be punished. This significantly differs from the tournament promotion city governance system. Under the tournament system, if the city fails to achieve its objectives designed by the central governance, its major officials will be punished [40,41]. However, the honorary title of ACS is not linked to the assessment of city officials. Therefore, cities can voluntarily participate in the evaluation activities, can decide not to participate, or even withdraw halfway. The city has strong autonomy [42]. 

Third, ACS implements a dynamic honorary titles management institution. The honorary title is not long-term; dynamic evaluation will be carried out during each assessment period. For cities that fail to meet the standard in the later review process, the superior department will revoke their honorary titles. Therefore, cities that participate in the ACS, will adopt a long-term view to take action in order to be awarded the honorary title. In the process of competing or maintaining honorary titles, cities will constantly invest resources to meet the requirements of the central government. In addition, the central government often improves the evaluation standards of some evaluation indicators and can also add some evaluation indicators, so cities always need to keep up with the highest benchmark in all indicators rather than only reaching the standard.

### 2.2. National Civilized City

The concept of the national civilized city stems from the transformation of China’s development strategy. In recent years, the Chinese central government has attached great importance to ecological civilization [33]. Centering on the evaluation of the major aspects of urban politics, the economy, culture, society, and ecological civilization, the Commission for the Development of Spiritual Civilization of the CPC Central Committee led and organized the selection of national civilized cities [33]. As a practical exploration by the Chinese government to promote the construction of ecological civilization [43], the national civilized city is a comprehensive honorary title reflecting the overall civilization level of cities in China. It is not only the highest-level and most valuable city honorary title but also the most competitive and the most difficult to establish city brand. Therefore, cities with the title of national civilized city can improve urban governance, improve the urban business environment, and further bring about direct and indirect economic and social benefits for the local people [44].

In 2003, the central government launched the program of evaluating and selecting the national civilized city and preliminarily issued selection criteria and application procedures. Then, the central government successively issued documents such as the national civilized city evaluation system, which explains meaning and requirements of the honorary title of the national civilized city. Based on the criteria, the first national civilized cities list was published in 2005. Then, the national civilized cities were selected and reviewed according to the procedure of “one big exam every three years and one small exam every year”. Therefore, the title of the national civilized city is not lifelong. Often, some cities are removed from the honorary title list, because they fail to pass their review. To maintain the honorary title, cities need to build a standard long-term mechanism to improve urban civilization. The number of cities that have been awarded the honorary title of the national civilized city increased from 8 in 2005 to 130 in 2020 [45]. 

Evaluation indicators of the national civilized city are extensively and dynamically updated. The 2021 edition of the national civilized city evaluation system covers 72 evaluation contents and 140 appraisal standards, involving government administration, the rule of law, the market, humanities, life, society, and ecology. The national civilized city evaluation also implements the negative list system and the one-vote veto system. The occurrence of major work safety accidents and Internet public opinion events will affect the selection score. The evaluation indicators of civilized cities will be dynamically updated every year. For instance, the 2021 version of the evaluation system added the social R&D expenditure ratio of total labor productivity indicators, which reflect urban innovation and business environment indicators.

The establishment of the national civilized city has three core characteristics: first, its status is high. Its guiding department is the Central Propaganda Department. The selected major officials of the city are awarded the title of the national civilized city and receive the award offered by the central government in Beijing. It is a great honor for the city and its officials. More importantly, in recent years, many provinces (autonomous regions and municipalities) have incorporated the achievements of the establishment of national civilized cities into the high-quality assessment system of local governments in a large proportion. In addition, the national civilized city is a typical kind of administrative competing system. The central government does not force local governments to declare their honorary title and encourages them to voluntarily participate in the establishment of national civilized cities. It emphasizes positive incentives, as well as providing an honorary title and political incentives. The central government will not punish city officials if the city fails to pass its examination.

### 2.3. Establishing National Civilized City and Urban Green Development

As a kind of administrative competing system, meeting the basic indicator’s standard is the premise of attaining the national civilized city title. The direct reason that the establishment of national civilized cities is conducive to urban green development lies in the fact that the evaluation index of the national civilized city consists of many environmental indicators, which are linked to environment protection, and industrial pollution emissions. These indicators include the environmental protection investment index, air pollution index (API), stable pollutant discharge compliance rate of key industrial enterprises, urban water environment function water quality compliance rate, forest coverage rate, black and odorous water body, energy consumption per unit GDP, industrial wastewater and waste gas treatment rate, air pollution index, smoke and dust control area compliance rate, and urban water area water quality compliance rate. Moreover, the national civilized city evaluation system implements a “one vote veto system” for major environmental pollution events. If a serious environmental accident occurs, the city will fail the examination. This encourages cities to monitor the ecological environment from multiple perspectives, such as air, water quality, greening, and investment.

While strengthening environmental protection, the evaluation system of national civilized cities also has high requirements for economic development. Especially in the early stage of the evaluation, it mandated that the per capita GDP of the city must exceed the national average development level for two consecutive years. The city cannot meet the evaluation standard by simply closing firms with high pollution levels; the only way to do this is to achieve compatible and balanced development between economic development and environmental protection. Therefore, all cities must reduce their environmental emissions per unit output, improve urban green total factor productivity, and promote urban green development while maintaining economic development. Research has shown that cities will spare no effort to launch various measures to improve the quality of development and optimize the development environment according to the requirements of the honorary title [33]. In sum, the central government takes honorary titles as rewards, measures and dynamically monitors the results of antinational civilized cities evaluation systems indicators, and encourages cities to boost green development, in order to fulfill the national green development strategy [46]. Therefore, the following hypothesis is proposed.

**H1.**  *The establishment of the national civilized city is positively related to urban green development*.

### 2.4. Mediators between Establishing National Civilized City and Urban Green Development

The evaluation of a national civilized city is a long-term dynamic evaluation process, which is also the premise for the effectiveness of the administrative competing system. After being awarded the honorary title of the national civilized city, all cities will regularly undergo stringent assessments carried out by the central government. Additionally, the central government will continuously and dynamically raise the assessment standards of relevant indicators in accordance with the development strategy of the state. For instance, as China pays an increasing amount of attention to its green development strategy, the national civilized evaluation system has raised the standard of the air PM2.5 index. With the increasing emphasis on innovation, the central government has newly set up “the proportion of R&D expenditure in GDP” index in the national civilized evaluation system. The long-term dynamic evaluation mechanism will urge local governments to step away from short-term vision and take long-term measures to promote urban green development. R&D is the cornerstone of urban green development. In the long term, technological innovation is the fundamental means to improve the efficiency of resource allocation. It can effectively reduce the energy consumption per unit output and is the basis of sustainable and green urban development [47]. Technological innovation will promote the upgrading of the urban industrial structure, which can gradually increase the number of clean and efficient industries, realize the continuous optimization of social resource allocation and the continuous improvement of production efficiency, and effectively support the improvement of green total factor productivity [18]. 

To meet the high standards of the national civilized city, local governments will increase subsidies for the green development of enterprises. The city will encourage enterprises to use government subsidies and increase R&D investment to innovate production technology, reduce energy consumption, speed up industrial upgrading, and fundamentally improve the city’s green total factor productivity. In the meantime, on one hand, local governments will formulate more strict regulation rules to urge enterprises to reduce emissions. On the other hand, local governments will invest much more financial resources and implement various environmental projects to protect the urban environment. Furthermore, the city will guide enterprises to raise their awareness of social responsibility, promote the construction of an ecosystem of corporate social responsibility and urge more enterprises to shoulder their due responsibilities in environmental protection. In a nutshell, the dynamic assessment practices of the national civilized city evaluation system promote local government to increase R&D investment, increase their financial resource investment, and optimize their industrial structure to boost urban green development. Therefore, we propose the following hypothesis:

**H2.**  *R&D investment, financial investment, and industrial structure are mediators between the establishment of the national civilized city and urban green development*.

## 3. Research Design

### 3.1. Model Specification

To investigate the effects of civilized city policies on urban green total factor productivity (GTFP), we employed the propensity scores matching (PSM) design [48] and difference-in-difference (DID) approach [49] based on the quasi-natural experiment of participating in the national civilized city award (NCCA) campaign. 

The setting of the classical DID model is shown in Equation (1):

(1) $$y_{it}=\beta_{0}+\beta_{1}treat_{i}\times post_{t}+\beta_{2}treat_{i}+\beta_{3}post_{t}+\alpha X_{it}+\varepsilon_{it}$$

where $y_{it}$, $X_{it}$, and $\varepsilon_{it}$ represent dependent variable, control variable, and random disturbance term of the ith city in the t period, respectively. $treat_{i}$ is a dummy variable: if i is a civilized city, it belongs to the treatment group, and the corresponding $treat_{i}$ takes a value of 1; if i is not a civilized city, then it belongs to the control group, and the corresponding $treat_{i}$ takes the value 0. $post_{t}$ is a dummy variable for policy implementation: before the policy implementation, $post_{t}$ takes a value of 0, and after the policy is implemented, $post_{t}$ takes a value of 1. $treat_{i}\times post_{t}$ is an interaction term between a group dummy variable and a policy dummy variable, and the coefficient $\beta_{1}$ reflects the net effect of policy implementation.

The Central Commission for Civilization of China announced six batches of national civilized cities in 2005, 2009, 2011, 2015, 2017, and 2020, respectively. Thus, the selection of civilized cities is a dynamic process, and the time nodes for each city to become a civilized city are different. Referring to the practice of the existing literature [30,33,50], this paper adopted the multi-period DID model to carry out the analysis, thus changing the form of Equation (1) into Equation (2):

(2) $$y_{it}=\beta_{0}+\beta_{1}civi\_city_{it}+\alpha X_{it}+\mu_{i}+\delta_{t}+\varepsilon_{it}$$

where $civi\_city_{it}$ is an interaction term between a group dummy variable and a policy dummy variable. If the ith city is selected as a civilized city in the T period, then $civi\_city_{it}$ takes a value of 1 when t≥T (the title of the ith city as a civilized city has not been revoked); otherwise, it is 0. The coefficient $\beta_{1}$ reflects the net effect of civilized city policy implementation on urban GTFP. $u_{i}$ and $\delta_{t}$ represent the individual fixed effect and time fixed effect, respectively. Other variables are defined in the same way as in Equation (1).

Since the civilized city selection is endogenous to the characteristics of the city, cities in the treatment group and the control group may not meet the balance test, thus affecting the estimation results. To solve this problem, this paper first adopted the PSM method to construct control samples with similar characteristics for selected civilized cities and then used the DID method for the estimation [50]. Specifically, according to whether a city has ever been selected as a civilized city, the sample selected as a civilized city is taken as the treatment group ($treat_{i}=1$), and the logit model is used to estimate Equation (3) to obtain the probability of a city being selected as a civilized city ($P_{c}$), which is taken as the propensity score. $X_{c}$ in Equation (3) is the assessment index that affects a city to be selected as a civilized city. Finally, the matched samples are used to estimate model Equation (2), and the estimation coefficient $\beta_{1}$ represents the net effect of civilized city policy implementation on urban GTFP.

(3) $$P_{c}=P_{i}\left\{L_{c}=1\left|X_{c}\right.\right\}=\phi (X_{c}^{\prime }\beta )$$

### 3.2. Variable Definition

#### 3.2.1. Dependent Variable

The dependent variable of this paper is GTFP. Referring to the practice of [51], our paper combined Malmquist’s TFP index and data envelopment analysis theory. In addition, we used Tone’s non-radial and non-angle slack-based measure (SBM) efficiency model [52,53] based on slack variables containing undesired output to construct the Malmquist productivity change index of intertemporal changes. The SBM efficiency model formula is as follows:

(4) $$\begin{array}{l} \min\rho^{*}=\frac{1+\frac{1}{t_{x}}\sum_{i=1}^{t_{x}}\frac{s_{i}^{x}}{x_{ix}}}{1-\frac{1}{t_{y}+t_{b}}(\sum_{r=1}^{t_{y}}\frac{s_{r}^{y}}{y_{rk}}+\sum_{l=1}^{t_{b}}\frac{s_{l}^{b}}{b_{lk}})}\\ s.t.\left\{\begin{array}{c} x_{ik}\geq \sum_{j=1,j\neq k}^{n}x_{ij}\lambda_{j}-s_{i}^{x}\\ b_{lk}\geq \sum_{j=1,j\neq k}^{n}x_{lj}\lambda_{j}-s_{l}^{b}\\ 1-\frac{1}{t_{y}+t_{b}}(\sum_{r=1}^{t_{y}}\frac{s_{r}^{y}}{y_{rk}}+\sum_{l=1}^{t_{b}}\frac{s_{l}^{b}}{b_{lk}})>0\\ s^{x},s^{y},s^{b},\lambda \geq 0;j=1,2,\ldots ,n(j\neq k) \end{array}\right. \end{array}$$

where λ is the weight vector; $x_{ik}$, $y_{rk}$, $b_{lk}$ represent the input variable, the expected output, and the undesired output, respectively. $s^{x}$, $s^{y}$, and $s^{b}$ represent the slack variables of the three types of elements, where $t_{x}$, $t_{y}$, and $t_{b}$ represent the three elements, respectively.

The Malmquist TFP index model is as follows:

(5) $$GTFP_{t}^{t+1}={\left[\frac{D^{t}(x^{t+1},y^{t+1},b^{t+1})}{D^{t}(x^{t},y^{t},b^{t})}\times \frac{D^{t+1}(x^{t+1},y^{t+1},b^{t+1})}{D^{t+1}(x^{t},y^{t},b^{t})}\right]}^{\frac{1}{2}}$$

where $GTFP_{t}^{t+1}$ represents the total factor productivity change index. If the value is greater than 1, the GTFP increases, and if it is less than 1, the GTFP decreases. Thus, the obtained Malmquist index reflects the growth of GTFP. This paper assumes that the green total factor growth rate of the base period is 1, and the annual multiplication of the Malmquist index represents the annual GTFP [54].

The input indicators selected in this paper include the three elements of labor, capital, and energy. Labor input is the number of employees at the end of the year. Capital input is measured by the total fixed assets investment with the classic perpetual inventory method [55]. The basic formula of capital input is as follows:

(6) $$K_{t}=(1-\delta )K_{t-1}+I_{t}$$

where $K_{t}$ represents the capital stock of period t, and δ represents the capital depreciation rate. Based on the practice of [52], the urban fixed assets depreciation rate δ is set to 9.6%, and the fixed investment amount of each city in 2006 is defined at 10% as the initial capital stock. Energy input is measured by the total electricity consumption. 

The output indicators in this paper include the expected output and undesired output. The GDP of each city is selected as the expected output. Due to the lack of GDP deflator at the city level, this paper adopts the GDP deflator of the province where the city is located to carry out the adjustment for the GDP of 281 cities at constant prices in 2000. Considering the outstanding urban air pollution caused by China’s industrialization process, industrial sulfur dioxide emissions and industrial soot emissions are adopted as the undesired output indicators.

Finally, this paper uses the DEAP2.1 program to obtain the GTFP (tfpch) of prefecture-level cities from 2000 to 2018.

#### 3.2.2. Independent Variable

The Independent variable is the national civilized city, and we used a dummy variable to indicate whether the policy was implemented. The value of $civi\_city_{it}$ is set to 1 from the year a prefecture-level city is awarded the title of “national civilized city”; if a civilized city loses its qualification in a certain year, then the value of $civi\_city_{it}$ is set to 0 until it regains the qualification of “national civilized city”; $civi\_city_{it}$ is set to 0 for both unselected cities and unselected years.

#### 3.2.3. Control Variable

Referring to the existing literature [56,57,58], this paper selects the factors affecting China’s green total factor productivity as control variables and includes six control variables in the regression analysis: financial development level (fin), foreign direct investment level (fdi), education investment (eduinv), human capital (edu), population size (lnpop), and level of urbanization (urban). fin is measured by the proportion of the balance of deposits and loans of financial institutions in urban GDP. fdi is measured by the proportion of foreign direct investment in urban GDP. eduinv is measured by the proportion of educational expenditure in urban financial expenditure. edu is measured by the proportion of ordinary undergraduate and junior college students in the total urban population. lnpop is measured by the log of the urban population. urban is measured by the proportion of the working population in the total urban population.

The descriptive statistics of the main variables are shown in Table 1.

### 3.3. Data Sources and Sample Selection

This paper selects the panel data of 281 Chinese prefecture-level cities from 2000 to 2018. All data used were obtained from the China Statistical Yearbook and China City Statistical Yearbook. In addition, some urban areas of Beijing, Shanghai, Tianjin, and Chongqing were selected as civilized cities (districts), but not the whole city was awarded the title of the civilized city, and the political and economic characteristics of the municipality are quite different from other cities, so this paper excludes the samples of these four municipalities.

## 4. Empirical Results

### 4.1. PSM Results

The premise of the DID method is that the treatment group and the control group have the same change trend before the policy implementation. If civilized cities pay more attention to green total factor development than other cities before the evaluation of civilized cities, the benchmark regression results of this paper would be unreliable. To avoid the selective error in the variation trend of the treatment group and the control group, the matching samples with propensity score were further analyzed. Specifically, this paper conducted a logit regression of the grouped dummy variables compared to the control variables (financial development level (fin), foreign direct investment level (fdi), education investment (eduinv), human capital (edu), population size (lnpop), and level of urbanization (urban) to obtain propensity matching scores using the “k-nearest neighbor matching method” (k = 2), and the cities with the closest propensity matching scores could be used as the control group. 

**Figure 1:**
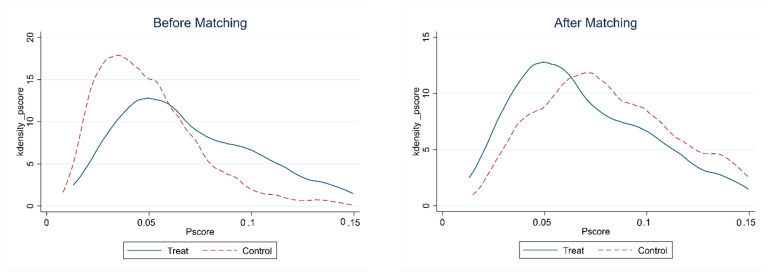
The probability density of the propensity scores.

Table 2 shows the balance test results based on the control variables. It was found that the *t*-statistic values of the covariate were not significant after matching; that is, the hypothesis that there was no significant systemic difference between the treatment group and the control group was accepted, and the value of bias was less than 10%, indicating that the matching process is effective. In addition, Figure 1 further shows the probability density of the propensity scores. Compared with the samples before matching, the probability density distribution of the propensity score values of the treatment group and the control group was closer after the nearest neighbor PSM matching, indicating that the matching effect in this paper is good. Therefore, both Table 2 and Figure 1 prove the rationality and reliability of using the PSM–DID method to analyze the research samples in this study.

### 4.2. Baseline Results

According to the benchmark regression, Equation (2), the impact of civilized city policy on the urban green total factor productivity was estimated, and the results are shown in Table 3. Table 3 reports the benchmark regression results of DID and PSM–DID. Both Models 1 and 2 represent the estimation results of the DID of unmatched samples (Model 1 does not contain control variables, and Model 2 contains control variables). Models 3 and 4 represent the PSM–DID estimation results of the experimental group and control group samples after matching (Model 3 does not contain control variables, and Model 4 contains control variables). As can be seen from Table 3, in the estimation results of the DID and PSM–DID, the coefficients of *civi_city* were significantly positive (0.141 in Model 1, 0.059 in Model 2, 0.094 in Model 3, and 0.072 in Model 4). Therefore, it can be proved that the “civilized city” policy is conducive to the improvement of the green total factor level of the city.

### 4.3. Robustness Test

#### 4.3.1. Parallel Trend Test

To intuitively observe the dynamic effects of civilized city policy, we referred to the research in [50]; a parallel trend test on the treatment group and the control group was carried out in this study. Referring to the program of the event study method, this paper changed the basic Equation (2) into the form of Equation (7):

(7) $$Y_{it}=\alpha_{0}+\sum_{k=1}^{5}\beta_{k}B_{k}+\sum_{k=1}^{5}\beta_{k}^{\prime }A_{k}+\alpha X_{it}+\mu_{i}+\delta_{t}+\varepsilon_{it}$$

where $B_{k}$ represents the k year before the selection of the civilized city, $A_{k}$ represents the k year after the selection of a civilized city, and other variables have the same meanings as in Equation (2). Therefore, $B_{k}$ and $\;A_{k}$ represent the dynamic effects of selected civilized cities, respectively. Based on the principle of the parallel trend test, it clear that the significance of $\;B_{k}$ and $A_{k}$ should be differentiated, the results of $B_{k}$ should be as insignificant as possible, and $A_{k}$ should be as significant as possible.

The results in Table 4 show that the estimated coefficients of the prior dummy variables (B1, B2, B3, B4, and B5) were not significant, while the estimated coefficients of the later dummy variables (A0, A1, and A2) were significant. These results are consistent with expectations, indicating that the research design of this paper meets the parallel trend hypothesis and the basic regression results are robust.

#### 4.3.2. Placebo Test

In this paper, counterfactual tests were used to conduct placebo tests. The traditional counterfactual test advances the policy time by one or two years to observe whether the policy effect is significant. Therefore, the counterfactual test advanced the implementation time of civilized city policy by one year (D1) and two years (D2), respectively, and the regression results are shown in Table 5. The coefficients of D1 in Models 1 and 2 or D2 in Models 3 and 4 were significant. Therefore, this proves that civilized city policy has a significant effect on the green total factor productivity of the city, and the baseline regression results are reliable.

## 5. Mechanism Analysis

According to the above baseline regression, it can be concluded that the civilized city policy can effectively improve urban green total factor productivity, but the specific mechanism of civilized city policy to improve urban green total factor productivity needs to be analyzed. Therefore, we further explore the influence of the civilized city policy on the urban green total factor productivity in three ways: science and R&D investment (tecinv), financial investment (govexp), and industrial structure (indstru) [12,31,45,56,59].

To verify the above mechanism, Wen and Ye [60] used the test method of the mediating effect to further expand Equations (8) and (9) based on Equation (2):

(8) $$M_{it}=\gamma_{0}+\gamma_{1}civi\_city_{it}+\gamma_{2}X_{it}+\mu_{i}+\delta_{t}+\varepsilon_{it}$$

(9) $$Y_{it}=\theta_{0}+\theta_{1}civi\_city_{it}+\theta_{2}M_{it}+\theta_{3}X_{it}+\mu_{i}+\delta_{t}+\varepsilon_{it}$$

where $M_{it}$ represents the mediating variables (tecinv, govexp, and indstru). tecinv is measured by the proportion of expenditure on science and technology in government expenditure. govexp is measured by the proportion of government expenditure in urban GDP. indstru is measured by the proportion of the service industry (China’s tertiary industry). $Y_{it}$ represents the dependent variable. The other variables have the same meanings as in Equation (2). 

Table 6 analyzes the effect mechanism of civilized city policy on urban green total factor productivity from the three aspects of R&D investment (tecinv), financial investment (govexp), and industrial structure (indstru), respectively. Models l and 2 in Table 6 explore the mediating effect of R&D investment (tecinv), Models 3 and 4 in Table 6 explore the mediating effect of financial investment (govexp), and Models 5 and 6 in Table 6 explore the mediating effect of industrial structure (indstru). All the coefficients of *civi_city* in Models 1 to 6 are significant, and the coefficients of tecinv, govexp, and indstru are significant in Table 6. Additionally, we also performed the Sobel test. All the results proved that the mediating effects of R&D investment (tecinv), financial investment (govexp), and industrial structure (indstru) are supported.

## 6. Heterogeneity Analysis

Based on the existing literature [52], the impact of the national civilized city policy on urban green development may be affected by the urban population size and economic scale. Therefore, this paper continues to conduct heterogeneous data analysis on the impact of the establishment of the national civilized city on urban green development.

### 6.1. Population Sizes Heterogeneity

According to the guidelines on adjusting the criteria for classifying cities in China, a city with a population of more than 10 million is a megacity, a city with a population of between 5 million and 10 million is a megacity, a city with a population of between 1 million and 5 million is a big city, and a city with a population of less than 1 million is a small or medium-sized city. To test whether civilized cities with different population sizes have an impact on the relationship between civilized cities and urban green total factor productivity, this paper divides cities according to the above criteria. As the number of cities with a population of less than 1 million and more than 10 million in the sample of this study is small, the group is divided into two groups according to the population size of more than or equal to 5 million and less than 5 million, and the analysis results are shown in Table 7. We can see that in the group of popsize = 0 (<500), the impact of civilized city policy on urban green total factor productivity is significantly positive (0.129), but in the group of popsize = 1 (≥500), the impact of civilized cities on urban green total factor productivity is nonsignificantly negative (−0.05). Therefore, the positive impact of the civilized city policy on urban green total factor productivity is greater with a smaller population.

### 6.2. Economic Scale Heterogeneity

In this paper, per capita GDP is taken as an indicator of the economic scale, and the samples are grouped by calculating the median. Cities are divided into two subsample sets: the high economic development (ecosize = 1) group and the low economic development (ecosize = 0) group. The regression results are shown in Table 7. As can be seen from the above table, the coefficients of *civi_city* in the ecosize = 0 group and ecosize = 1 group are −0.011 (insignificant) and 0.098 (significant), respectively. This shows that civilized city policy has a more obvious promoting effect on the green total factor productivity of cities with high economic development.

### 6.3. Summary

First, the above results show that the national civilized city policy is positively related to the GTFP, which means the establishment of a national civilized city can significantly improve urban green development; thus, H1 is supported.

Second, the above results prove that the mediating effects of R&D investment, financial investment, and the industrial structure in the relationship between the establishment of the national civilized city and urban green development are significant; thus, H2 is supported.

Last, the results of the heterogeneity analysis show that the positive impact of national civilized city policy on urban GTFP is significant and increases with a smaller urban population or higher urban economic development. 

The hypothesis test results are shown in Table 8.

## 7. Conclusions

Although the national civilized city policy does not provide economic benefits to local governments; cannot enhance the promotion probability of city chief officials; and may cause public opinion events in the selection process, which will have a negative impact on the city, the enthusiasm of all cities to participate in the establishment of the national civilized city is still very high. The fundamental reason for this may be that the establishment of a national civilized city can force local governments to issue more stringent environmental governance rules, increase R&D investment, accelerate industrial upgrading, promote green urban development, build a highly civilized city, gain a good reputation among government officials, and gain an improved political reputation.

### 7.1. Theoretical Implications

First, the applicability of the administrative competing system in the context of China is verified. As a typical practice of an administrative competing system, the establishment of the national civilized city encourages local governments to initiate competition in accordance with their conditions and respond to the central government’s initiative. The research shows that the practice of establishing the administrative competing system of the national civilized city has high applicability in the field of public management in China, provides a new choice of policy tool for local government management other than “pressure” and “the contract system”, and enriches the research on the urban governance system with Chinese characteristics.

Second, the core conditions for the administrative competing system to play a role are analyzed. The premise of the national civilized city in creating a developed governance effect is that it can provide political incentives for local governments and an honorary title. The key to this is superior departments dynamically setting performance targets and standards, performing ongoing assessments, regularly applying pressure, reversing the transmission effect, encouraging local governments to practice integration, and promoting urban comprehensive management ability. This shows that the precondition for the administrative competing system to play a role is stimulating the bidding motivation of local governments and continuously optimizing the monitoring of bidding targets and regular assessment, which helps to deepen the theoretical understanding of the core elements of the administrative competing system.

Third, this study analyzes the internal mechanism of building civilized cities and promoting urban green development. In this paper, with the PSM and DID methods, through the comparative analysis between the green development of the national civilized city and the unnational civilized city, it is revealed that the R&D investment, financial investment, and industrial structure optimization create the main path to a national civilized city, affecting the development of the precise evaluation of the national civilized city to create a green growth-promoting effect. This provides a deeper evaluation of the national civilized city establishment policy.

### 7.2. Managerial Implications

First, we need to further promote the building of civilized cities nationwide to facilitate green urban development. Now, some have doubts about the establishment and selection of civilized cities. However, this study suggests that the establishment of a national civilized city can effectively promote urban green development. Relevant departments should continue to strengthen and innovate establishment activities to better motive the local government to attain the national civilized city honorary title and promote citizen participation in the national civilized city.

Second, the government should increase the proportion of environmental management indicators in the evaluation of the national civilized city, and give full play to the function of the policy tool to create e national civilized cities. The government should continue to improve regular and comprehensive assessments, as well as veto, retention, and exit mechanisms, which are the keys to the smooth running of the national civilized city. The local government should be urged to pay attention, invest financial resources in the field of urban environmental management and green development, and promote the development of high-quality cities.

Third, a heterogeneity analysis showed that, for cities with a higher level of economic development, the establishment of the national civilized city has a greater impact on urban green development. This indicates that in the process of the evaluation of the national civilized city, the Central Committee of Civilization should fully consider the difference in a city’s resource endowment, set up the environmental evaluation index and weight according to local conditions, and better play the role of evaluation and recognition tool for the national civilized city.

Fourth, the administrative competing system is a unique mode of urban governance in China, which is rooted in the historical reality and political system of China. Compared to many one-size-fits-all incentive and pressure systems in other countries, it can provide a Chinese influence in other countries’ urban management [37]. These experiences can be summarized from three perspectives: the position of the design subject of the competing project, the management mechanism of the execution subject of the competing project and the service object of the competing project. First, the competing project design subject should have high political authority to anchor the long-term attention of the city. Second, the execution subject of the competing project should build a dynamic management mechanism to improve the overall governance ability of the government. Third, the service object of the competing project must mobilize all parties to participate in coordination, with people at the center.

### 7.3. Limitations and Future Research Directions

First, the influence of the establishment of a national civilized city on urban green development will be moderated by a variety of factors, such as competition among cities, the urban administrative level, and the region where the city is located. This paper does not conduct an in-depth analysis of the moderating effect of these variables on the relationship between civilized cities and urban green development in China. In the future, the threshold effect of urban spatial competition, urban human capital, and other variables can be analyzed.

Second, the input and output indicators of urban green total factor productivity need to be further refined to improve the robustness of research conclusions. The establishment of a national civilized city is a major livelihood project, which provides citizens with greater public services and improves people’s sense of achievement. In the future, the influence of the establishment of a national civilized city on the equalization of urban public services should be further discussed.

## Figures and Tables

**Table 1:** Descriptive statistics.

Variable	Mean	Std	Min	Max
tfpch	1.143	0.669	0.093	12.569
$civi\_city_{it}$	0.106	0.308	0.000	1.000
fin	1.233	1.078	0.075	24.800
fdi	0.029	0.095	0.000	2.647
eduinv	0.052	0.085	0.000	2.502
edu	1.412	2.118	0.000	35.022
lnpop	5.823	0.686	2.77	9.315
urban	0.116	0.110	0.003	1.473

Notes: Std—represents standard deviations.

**Table 2:** Balance test.

Variable	Mean			Test	
	Treated	Control	Bias (%)	*t*	*p* > |*t*|
*fin*	1.781	1.748	2.900	0.360	0.715
*fdi*	0.034	0.033	1.500	0.470	0.638
*eduinv*	0.050	0.046	4.900	1.130	0.260
*edu*	1.891	1.761	7.300	0.930	0.354
ln*pop*	5.992	6.004	−1.700	−0.270	0.788
*urban*	0.223	0.220	1.900	0.300	0.764

**Table 3:** Baseline results.

Variable	tf*pch*			
	Model 1 DID	Model 2 DID	Model 3 PSM-DID	Model 4 PSM-DID
*civi_city*	0.141 *** (0.03)	0.059 ** (0.024)	0.094 ** (0.038)	0.072 ** (0.029)
*fin*		0.442 *** (0.006)		0.483 *** (0.008)
*fdi*		0.336 *** (0.07)		0.309 *** (0.081)
*eduinv*		−0.36 *** (0.078)		−1.060 *** (0.171)
*edu*		−0.066 *** (0.004)		−0.066 *** (0.004)
ln*pop*		0.039 *** (0.011)		0.027 * (0.015)
*urban*		−0.337 *** (0.077)		−0.489 *** (0.095)
Constant	1.128 *** (0.01)	0.499 *** (0.065)	1.176 *** (0.016)	0.489 *** (0.095)
Year	Yes	Yes	Yes	Yes
City	Yes	Yes	Yes	Yes
R^2^	0.004	0.48	0.002	0.538
F	22.33	694.44	6.12	517.10
N	5268	5268	3100	3100

Notes: *, **, and *** indicate significance levels of 10%, 5%, and 1%, respectively; standard deviations are shown in parentheses.

**Table 4:** Parallel trend test.

tf*pch*	Coef.	Std. Err.	*t*	*p* > |*t*|	(95% Conf. Interval)	
B5	−0.02	0.05	−0.51	0.61	−0.12	0.07
B4	−0.04	0.05	−0.80	0.42	−0.13	0.05
B3	−0.02	0.05	−0.34	0.74	−0.11	0.08
B2	−0.03	0.05	−0.55	0.58	−0.12	0.07
B1	−0.06	0.05	−1.20	0.23	−0.15	0.04
A0	0.16	0.05	3.21	0.00	0.06	0.25
A1	−0.09	0.05	−1.85	0.07	−0.18	0.01
A2	0.22	0.06	3.70	0.00	0.10	0.34
A3	−0.09	0.06	−1.50	0.13	−0.21	0.03
A4	0.00	0.08	−0.06	0.95	−0.17	0.16
A5	−0.13	0.08	−1.63	0.10	−0.29	0.03
Control variable	yes					
Year	yes					
City	yes					
R^2^	0.3855					
F	192.48					

**Table 5:** Placebo test.

Variable	tf*pch*			
	Model 1 DID	Model 2 DID	Model 3 PSM-DID	Model 4 PSM-DID
D1	−0.066 (0.03)		−0.053 (0.055)	
D2		−0.032 (0.337)		−0.008 (0.055)
Control variable	Yes	Yes	Yes	Yes
Year	Yes	Yes	Yes	Yes
City	Yes	Yes	Yes	Yes
R^2^	0.477	0.477	0.538	0.537
F	76.73	76.66	515.45	515.18
N	5326	5326	3100	3100

**Table 6:** Mechanism analysis results.

Variable	te*cinv*	tf*pch*	*gov*exp	tf*pch*	*indstru*	tf*pch*
	Model 1	Model 2	Model 3	Model 4	Model 5	Model 6
*civi_city*	**0.011** *** (0.003)	**0.054** ** (0.024)	**0.036** *** (0.012)	**0.256** *** (0.067)	**0.023** *** (0.004)	**0.041** * (0.022)
te*cinv*		**0.404** *** (0.112)				
*gov*exp				**0.84** *** (0.307)		
*indstru*						**1.082** *** (0.083)
*fin*	−0.002 * (0.001)	0.442 *** (0.006)	0.163 *** (0.011)	0.303 (0.038)	0.027 *** (0.001)	0.399 *** (0.007)
*fdi*	−0.009 (0.009)	0.34 *** (0.07)	0.047 ** (0.022)	0.251 (0.154)	0.023 ** (0.01)	0.366 *** (0.06)
*eduinv*	−0.729 *** (0.01)	−0.066 (0.113)	−0.46 *** (0.058)	0.088 (0.148)	0.119 *** (0.011)	−0.11 (0.067)
*edu1*	−0.002 *** (0.000)	−0.065 *** (0.004)	−0.008 *** (0.002)	−0.047 *** (0.007)	0.016 *** (0.01)	−0.035 *** (0.004)
ln*pop*	0.019 *** (0.001)	0.033 *** (0.011)	−0.026 *** (0.009)	0.061 *** (0.015)	0.009 *** (0.002)	0.031 *** (0.009)
*urban*	−0.016 * (0.009)	−0.331 *** (0.076)	−0.169 *** (0.056)	−0.172 (0.218)	0.017 (0.011)	−0.287 *** (0.067)
Constant	0.072 *** (0.008)	0.47 *** (0.065)	0.173 *** (0.051)	0.339 *** (0.100)	0.255 *** (0.01)	0.92 *** (0.06)
Year	Yes	Yes	Yes	Yes	Yes	Yes
City	Yes	Yes	Yes	Yes	Yes	Yes
R^2^	0.536	0.482	0.641	0.508	0.356	0.384
F	867.732	610.622	564.993	597.858	399.252	3146.117
N	5268	5268	5268	5268	5268	5268

Notes: *, **, and *** indicate significance levels of 10%, 5%, and 1%, respectively; standard deviations are shown in parentheses.

**Table 7:** Heterogeneity analysis.

Variable	tf*pch*		tf*pch*	
	Popsize = 0 (<500)	Popsize = 1 (≥500)	Ecosize = 0	Ecosize = 1
*civi_city*	**0.129** *** (0.034)	**−0.05** (0.032)	−0.011 (0.032)	0.098 *** (0.023)
*fin*	0.413 *** (0.008)	0.517 *** (0.01)	0.543 *** (0.007)	0.209 *** (0.013)
*fdi*	0.314 *** (0.08)	0.475 *** (0.156)	0.105 (0.082)	0.732 *** (0.118)
*eduinv*	−0.425 *** (0.094)	−0.113 (0.138)	−0.427 *** (0.092)	−0.029 (0.136)
*edu1*	−0.063 (0.005)	−0.068 *** (0.006)	−0.168 *** (0.024)	−0.035 *** (0.004)
ln*pop*	0.061 *** (0.016)	−0.041 (0.04)	−0.045 (0.048)	0.022 * (0.013)
*urban*	−0.357 *** (0.087)	−0.305 (0.204)	0.785 *** (0.274)	−0.249 *** (0.076)
Constant	0.421 *** (0.094)	0.924 *** (0.26)	0.886 *** (0.29)	0.795 *** (0.076)
Year	Yes	Yes	Yes	Yes
City	Yes	Yes	Yes	Yes
R^2^	0.433	0.608	0.595	0.129
F	2734.362	2597.569	5627.48	366.416
N	3583	1685	2873	2395

Notes: *, and *** indicate significance levels of 10%, and 1%, respectively; standard deviations are shown in parentheses.

**Table 8:** Hypothesis test results.

Hypothesis	Hypothesis Context	Test Results
**H1**	*The establishment of the national civilized city is positively related to urban green development*.	Supported
**H2**	*(1) R&D investment is a mediator between the establishment of the national civilized city and urban green development.*	Supported
	*(2) Financial investment is a mediator between the establishment of the national civilized city and urban green development.*	Supported
	*(3) Industrial structure is a mediator between the establishment of the national civilized city and urban green development.*	Supported
